# Electromagnetic neuromodulation for chronic pain: historical context, contemporary modalities, and evidence gaps

**DOI:** 10.3389/fpain.2026.1901466

**Published:** 2026-08-18

**Authors:** Laura Dipietro, Ciro Ramos-Estebanez, Paola Gonzalez-Mego, Anna Carolyna Gianlorenco, Arisara Wongtanawat, Christopher Robinson, Salim M. Hayek, Felipe Fregni, Timothy Wagner

**Affiliations:** 1 Highland Instruments, Cambridge, MA, United States; 2 Departments of Neurology and Rehabilitation, and Neurosurgery, University of Illinois at Chicago, Chicago, IL, United States; 3 Spaulding Neuromodulation Center, Spaulding Rehabilitation Hospital and Massachusetts General Hospital, Boston, MA, United States; 4 Department of Physical Medicine and Rehabilitation, Harvard Medical School, Boston, MA, United States; 5 Division of Pain, Department of Anesthesiology and Critical Care Medicine, Johns Hopkins University School of Medicine, Baltimore, MD, United States; 6 Division of Pain Medicine, University Hospitals Cleveland Medical Center, Cleveland, OH, United States; 7 Department of Anesthesiology, Case Western Reserve University, Cleveland, OH, United States; 8 Harvard-MIT Program in Health Sciences and Technology, Cambridge, MA, United States

**Keywords:** brain stimulation, central sensitization, chronic pain, neuromodulation, peripheral nerve stimulation, spinal cord stimulation, transcranial direct current stimulation, transcranial magnetic stimulation

## Abstract

Electromagnetic neuromodulation is increasingly used as an adjunct or alternative to pharmacologic therapy for pain, with several approaches cleared for select indications and many others under active investigation. We review implanted neuromodulation approaches (intracranial and spinal), vagal neuromodulation, noninvasive central/cranial neuromodulation, peripheral neuromodulation, and hybrid/multimodal approaches, with a primary focus on chronic pain management and brief coverage of selected acute-use indications (e.g., primary headache disorders) where relevant. Beyond summarizing individual modalities, we highlight the recurring patterns that have shaped the evolution of pain neuromodulation. For each modality, we summarize putative mechanism of action, typical stimulation paradigms, and key strengths and limitations of the clinical evidence, emphasizing sources of heterogeneity that complicate cross-study comparison and translation. We conclude by outlining priorities for the field, including more rigorous trial design, standardized reporting of stimulation parameters and outcomes, and clearer indication-specific evidence to support clinical translation.

## 1 Introduction: from early electroanalgesia to modern neuromodulation

Pharmacologic therapy is widely used to treat chronic pain, but its clinical utility is often limited by incomplete efficacy, adverse effects, and, for some agents, risks of misuse and dependence. In response to these limitations, electrical and magnetic neuromodulation approaches have emerged as adjunctive or alternative strategies for pain management, with several technologies cleared or approved by the Food and Drug Administration (FDA) for select pain indications. This review focuses on invasive and noninvasive neuromodulation methods, in which the therapeutic stimulus is principally electrical or magnetic, broadly considered here under the umbrella of electromagnetic neuromodulation, with emphasis on their application to chronic pain.

The first recorded use of electricity in medicine is commonly attributed to Scribonius Largus, who in Compositiones (around AD 47) described the use of a live torpedo fish to treat gout pain and headache (1, 2). The therapeutic use of electric fish persisted into late antiquity, and Galen also discussed the torpedo fish in relation to pain relief (3). Later developments replaced animal-derived electricity with man-made electrical sources. In the 1700s, Leyden jars (4), an early precursor to batteries, were explored as an electrical source for therapeutic pain relief (5). Work by Galvani and Aldini also helped establish electrical stimulation as an experimental tool for probing neuromuscular and brain function (6–8). In the late 1800s and early 1900s, electrical instruments were deployed for a variety of pain therapies, culminating in a chapter on “Electrical Analgesia, Sleep, and Resuscitation” in Gwathmey and Baskerville's 1914 “Anesthesia” textbook (9).

Electrical stimulation for pain entered the modern era in the late 1960s, amid broader interest in transcranial “electrical anesthesia,” when Reynolds demonstrated that microstimulation of the rat midbrain central gray could produce analgesia (10). Human case reports soon followed, describing analgesic effects of stimulation targeting the internal capsule (IC) (11) and periventricular/periaqueductal gray (PVG/PAG) (12, 13). The advent of modern noninvasive brain stimulation further expanded the field. Following the demonstration of noninvasive magnetic stimulation of human motor cortex in 1985 (14), repetitive transcranial magnetic stimulation (rTMS) protocols were subsequently explored as analgesic interventions, initially emphasizing the primary motor cortex (M1) and later extending to prefrontal and parietal targets to engage both sensory–discriminative and cognitive–affective dimensions of pain.

Conceptual advances in pain neuroscience also shaped device development: the gate control theory of pain proposed by Melzack and Wall (15) provided a mechanistic framework for why activation of large-diameter afferents might inhibit nociceptive transmission, helping motivate the clinical uptake of peripheral and spinal stimulation approaches. Moreover, these ideas, together with evolving concepts of descending pain modulation and, later, central sensitization, supported interest in cortical and supraspinal targets for analgesia (16–18). These developments helped establish electromagnetic neuromodulation as a broader strategy for pain management, with several technologies cleared for specific chronic pain conditions and acute-use applications, most prominently primary headache disorders.

Pain processing is distributed across multiple interacting levels of the nervous system rather than being confined to a single anatomical site. Peripheral noxious stimuli are transduced by nociceptors and conveyed primarily by thinly myelinated Aδ and unmyelinated C fibers (19) to the dorsal horn, where segmental gating, convergence of nociceptive and non-nociceptive inputs, and activity-dependent sensitization shape onward transmission (15, 16, 18, 20–22). Ascending pathways relay signals to thalamic, somatosensory, insular, cingulate, and related limbic-cortical regions involved in the sensory, affective, attentional, and evaluative dimensions of pain (23–26). In parallel, descending modulatory systems involving structures such as the PAG, rostral ventromedial medulla (RVM), and brainstem monoaminergic pathways can suppress or facilitate nociceptive processing (27, 28). In chronic pain states, maladaptive plasticity may emerge across this system, including central sensitization, impaired inhibitory control, abnormal facilitation, and altered network-level processing (29). This framework provides a rationale for organizing electromagnetic neuromodulation strategies according to their primary anatomical targets, while recognizing that many ultimately exert distributed downstream effects across the broader pain network.

**Figure 1:**
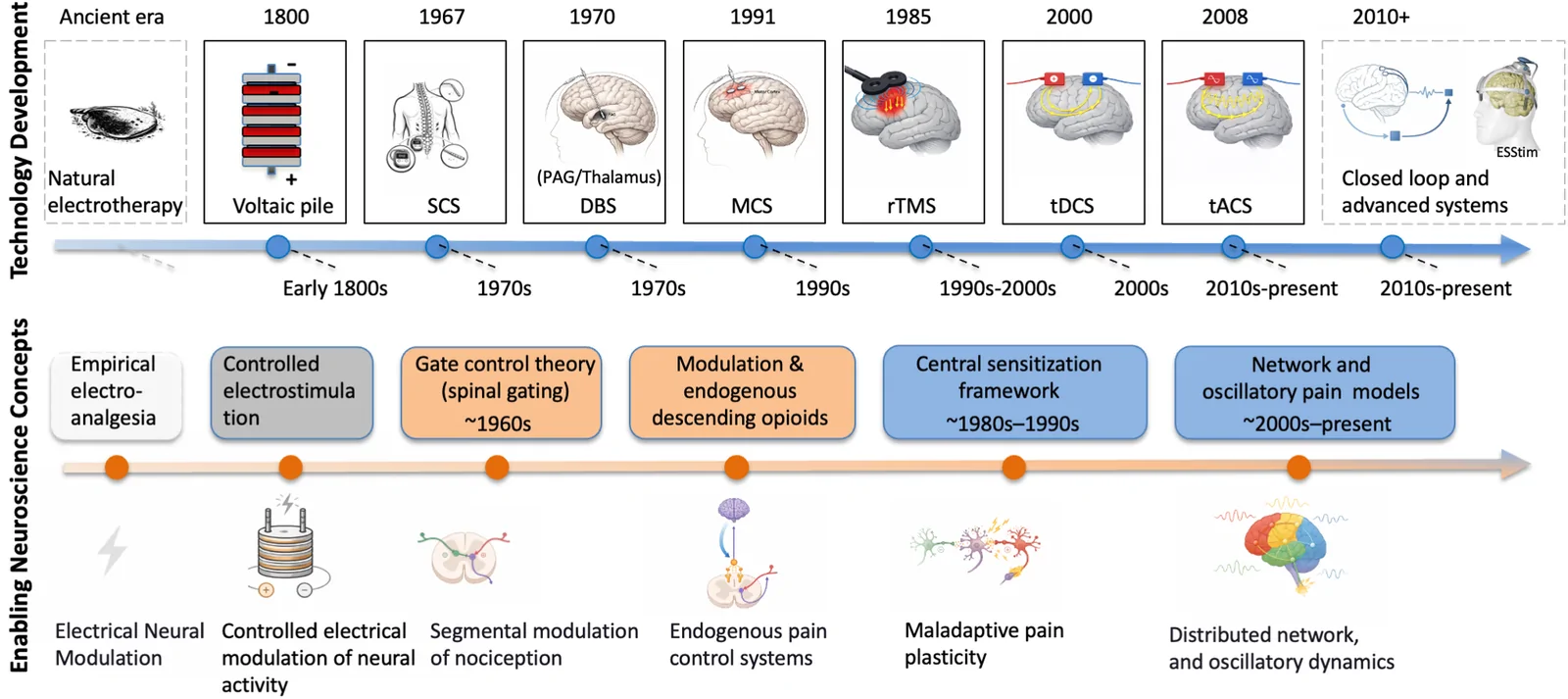
Timeline of electromagnetic neuromodulation for pain, showing major technological developments (top) and corresponding advances in neuroscience (bottom). The figure highlights the progression from empirical electro-analgesia to contemporary neuromodulation approaches, alongside evolving mechanistic frameworks of pain modulation.

Pain can further be classified in several ways, including by duration (acute or chronic) and by dominant mechanism (nociceptive, neuropathic, or central), although these categories often overlap in clinical practice. We use the term chronic to refer to pain that persists long enough to permit maladaptive changes within the nervous system, including central sensitization (29). Mechanistically, however, ample evidence shows that pain definitions are not mutually exclusive and instead share overlapping features; moreover, shared pathophysiology may add to the overlap of pain syndromes (18, 29–32). As a result, clinicians frequently encounter patients with mixed mechanisms for pain. Specifically, conditions originating in the periphery can induce central sensitization (16, 18, 32), i.e., neuroplastic changes associated with remodeling, reorganization, and altered neural excitability of the central nervous system (26, 33–38). Within this overlapping mechanistic framework, neuromodulation strategies can be considered according to where they act along the pain pathway: in the periphery [e.g., peripheral nerve stimulation (PNS)], along ascending pathways [e.g., TENS or spinal cord stimulation (SCS)], indirectly through cortical network effects [e.g., motor cortex stimulation (MCS) over M1, thought to ultimately influence the PAG and RVM], and/or directly within the brain [e.g., deep brain stimulation (DBS) implanted in the PAG]. Thus, as part of this review, we also consider the hypothesized mechanisms of action of each stimulation modality, although these mechanisms remain incompletely understood and, in many cases, debated. Figure 1 summarizes major conceptual milestones alongside key device innovations in electromagnetic neuromodulation.

**Figure 2:**
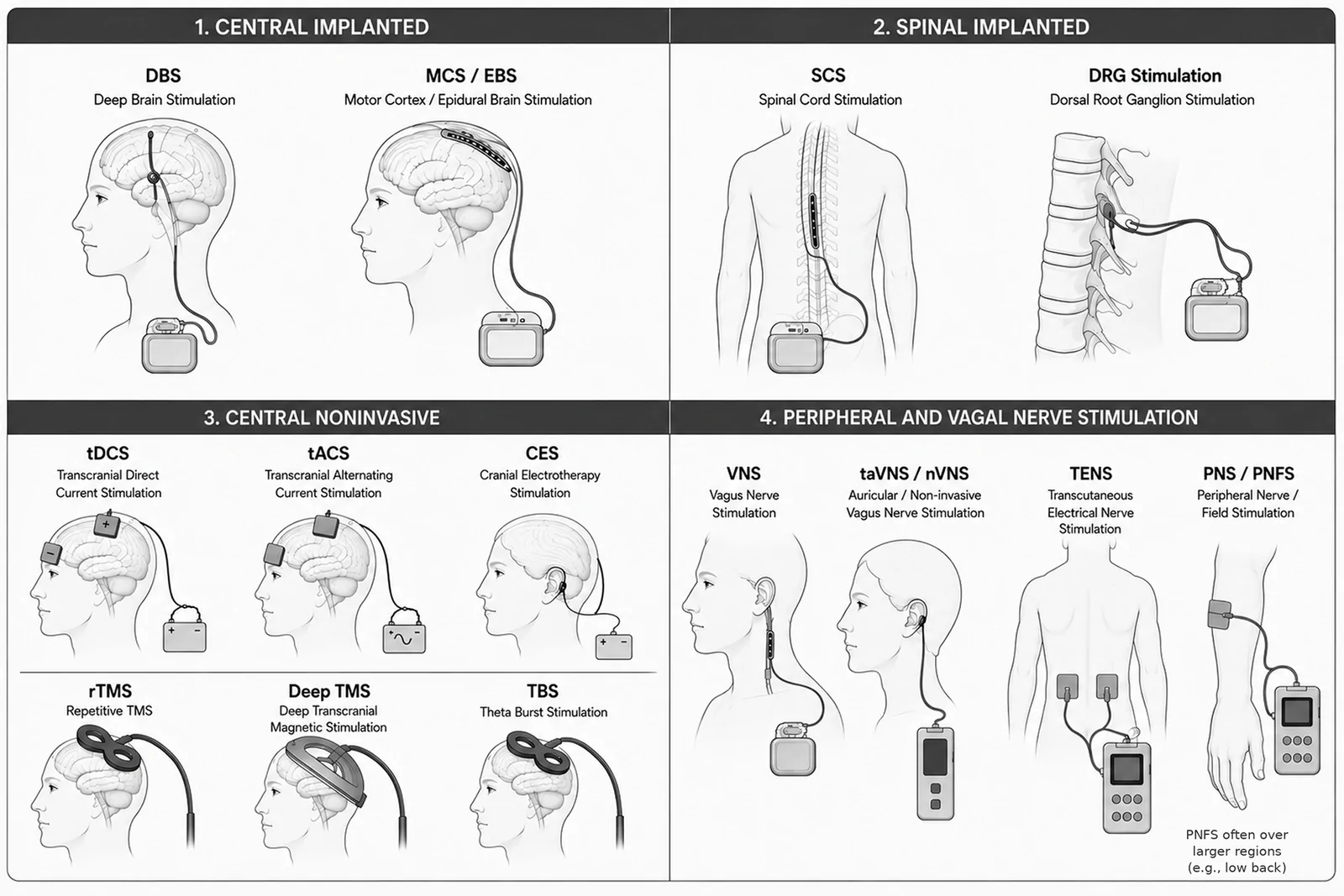
Schematic overview of major electromagnetic neuromodulation modalities for pain, organized by anatomical target and mode of delivery.

The sections below are organized by modality and principal anatomical target, while also situating each approach within its historical development and current evidence base. Figure 2 provides a schematic overview of the principal electromagnetic neuromodulation modalities discussed below, organized by anatomical target and mode of delivery.

Given the breadth of the field, this review integrates modalities with differing levels of clinical maturity, evidence strength, and regulatory status to provide a cross-modality perspective on how electromagnetic neuromodulation is currently applied and evolving in pain medicine. We emphasize a recurring pattern in which early mechanistic models drive technological innovation and subsequent clinical adoption, followed by heterogeneous outcomes that stimulate an iterative process of updated knowledge of pain physiology and subsequent device optimization towards better clinical outcomes. In addition to summarizing the historical development, mechanisms, regulatory status, and clinical applications of these modalities, this review highlights evidence gaps that limit interpretation and translation of the current literature. By synthesizing these limitations across electromagnetic neuromodulation approaches, we aim to clarify where these technologies have shown clinical promise and where stronger evidence is needed.

### 1.1 Scope and literature search strategy

This narrative review examines the historical development, current clinical use, and remaining evidence gaps in electromagnetic neuromodulation for pain management, with primary emphasis on chronic pain. The review includes implanted and noninvasive approaches acting across the peripheral, spinal, autonomic, cranial/transcranial, cortical, and intracranial levels of the nervous system, as well as selected combined approaches. Selected short-term or acute-use paradigms, such as transcutaneous electrical nerve stimulation (TENS) and noninvasive vagal nerve stimulation (nVNS) for acute migraine and cluster headache episodes, are briefly noted when they help contextualize the development or regulatory status of technologies also relevant to chronic pain. Because several electromagnetic neuromodulation devices carry regulatory indications spanning both chronic pain and episodic headache disorders, FDA-cleared or approved uses are summarized separately for chronic and acute/episodic indications in Tables 1, 2. The sections that follow, together with Table 3, address the broader literature, including applications beyond current regulatory labeling.

**Table 1:** FDA-cleared/approved electromagnetic neuromodulation devices for chronic pain.

Modality	FDA decision date	FDA-authorized use (summary)^a^	Applicant	Device	Authorization type	Source
SCS	11/30/84	Chronic intractable pain of the trunk and/or limbs	Medtronic	Multiple generations via supplements of PMA P840001; legacy: Itrel/Itrel 3/Synergy	PMA	FDA PMA P840001
SCS	11/21/01	Chronic intractable pain of the trunk and/or limbs	Advanced Neuromodulation Systems (ANS); later St. Jude Medical; now Abbott	Multiple generations via supplements of PMA P010032; legacy: Genesis/Eon/Prodigy/Proclaim (incl. XR/Plus)/Eterna	PMA	FDA PMA P010032
SCS	4/27/04	Chronic intractable pain of the trunk and/or limbs	Advanced Bionics; now Boston Scientific	Multiple generations via supplements of PMA P030017; legacy/platforms include Precision/Precision Spectra/Precision Novi/Precision Montage (MRI)/Spectra WaveWriter/WaveWriter Alpha (Prime)	PMA	FDA PMA P030017
SCS	5/8/15	Chronic intractable pain of the trunk and/or limbs	Nevro Corporation	Multiple generations via supplements of PMA P130022; platforms include: Senza/Senza II/Senza Omnia (10 kHz SCS)	PMA	FDA PMA P130022
SCS	11/20/15	Chronic intractable pain of the trunk and/or limbs	Nuvectra Corporation	Multiple generations via supplements of PMA P130028; platform: Algovita™ SCS System (legacy product line)	PMA	FDA PMA P130028
SCS	2/28/22	Chronic intractable pain of the trunk and/or limbs	Saluda Medical	Multiple generations via supplements of PMA P190002; platform: Evoke® SCS System (evoked compound action potential (ECAP) controlled closed-loop)	PMA	FDA PMA P190002
SCS	3/31/23	Chronic intractable pain in the trunk and/or limbs	Biotronik Nro, Inc	Multiple generations via supplements of PMA P210037; platform: Prospera™ SCS System	PMA	FDA PMA P210037
DRG	2/11/16	CRPS I/II	Abbott Medical	Axium Neurostimulator System; later supplements under P150004 include Proclaim DRG devices	PMA	FDA PMA P150004
Implanted PNS	2/20/15	Severe intractable chronic pain of peripheral nerve origin (adjunct to other therapies)	Bioness, Inc	StimRouter Neuromodulation System	510(k)	FDA 510(k) K142432
Implanted PNS	3/29/19	Chronic, intractable pain of peripheral nerve origin	Nalu Medical, Inc	Nalu Neurostimulation System	510(k)	FDA 510(k) K183579
Implanted PNS	6/20/24	Chronic, intractable pain of peripheral nerve origin	Curonix	Freedom PNS System	510(k)	FDA 510(k) K233162
Implanted PNS	8/27/21	Severe intractable chronic pain of peripheral nerve origin	Neuspera Medical, Inc	Neuspera Neurostimulation System	510(k)	FDA 510(k) K202781
Implanted PNS	3/11/16	Severe intractable chronic pain of peripheral nerve origin	StimQ, LLC	StimQ	510(k)	FDA 510(k) K152178
Restorative neurostimulation (implanted neuromuscular stimulation—multifidus)	6/16/20	Intractable chronic low back pain associated with multifidus muscle dysfunction (adults; failed therapy; not candidates for spine surgery)	Mainstay Medical Limited	ReActiv8	PMA	FDA PMA P190021
TENS	8/30/24	Chronic intractable pain; post-traumatic and post-surgical pain	TensCare Ltd	Unipro	510(k)	FDA 510(k) K232441
TENS	2/23/24	Chronic intractable pain; temporary relief of musculoskeletal pain (sore/aching muscles)	Hinge Health, Inc	ENSO (Model 2)	510(k)	FDA 510(k) K233784

Representative devices organized by modality and labeled indication, spanning invasive spinal neuromodulation (SCS, DRG stimulation), peripheral neuromodulation (implanted PNS; restorative multifidus neurostimulation), and transcutaneous approaches (TENS) with chronic pain labeling. “Authorization type” reflects the primary U.S. regulatory pathway [PMA vs 510(k)]; device generations are often updated via supplements/iterations under the same regulatory file. Applicant names reflect the original PMA holder where historically relevant, with current successor companies noted when applicable. This table is not intended to be exhaustive, particularly for categories with numerous substantially equivalent 510(k) devices (e.g., TENS).

a Use statements were summarized and harmonized from FDA marketing authorization documents for comparability and may not reproduce the exact original wording.

**Table 2:** FDA-cleared/approved electromagnetic neuromodulation devices for acute pain indications.

Modality	FDA decision date	FDA-authorized use (summary)^a^	Applicant	Device	Authorization type	Source	Notes
TENS	10/13/16	Temporary relief of sore/aching muscle pain (strain-related musculoskeletal pain)	Counter Scientific Development (GZ) Ltd	OTC TENS Device, Model PTS-IV	510(k)	FDA 510(k) K161537	
TENS	6/3/16	Adjunctive treatment for post-surgical and post-trauma acute pain	Djo, LLC	Strive	510(k)	FDA 510(k) K153704	Also indicated for chronic intractable pain and arthritis pain
TENS	12/6/11	Acute pain; post-surgical pain	Electronic Waveform Lab, Inc	H-Wave	510(k)	FDA 510(k) K112485	Also indicated for chronic pain
TENS	3/27/24	Post-surgical and post-traumatic acute pain	Wuxi Jiajian Medical Instrument Co., Ltd	TENS WMPS2-1	510(k)	FDA 510(k) K231425	Also indicated for chronic intractable pain
TENS component of Combo Stimulator	7/29/11	Symptomatic relief of acute post-surgical and posttraumatic pain	Handelshaus Dittman GmbH	ETG 255 Combo Stimulator/TEN 260 Stimulator	510(k)	FDA 510(k) K110390	Also indicated for chronic intractable pain
Temporary percutaneous PNS	1/25/23	Post-operative and post-traumatic acute pain; up to 60 days	SPR Therapeutics, Inc	SPRINT	510(k)	FDA 510(k) K223306	Also indicated for chronic, intractable pain
Auricular percutaneous electrical nerve stimulation (PENS)	1/31/22	Post-operative pain following cesarean section delivery; up to 3 days	DyAnsys, Inc	Primary Relief	510(k)	FDA 510(k) K213188	
TMS (single-pulse, sTMS)	12/13/13	acute treatment of pain associated with migraine headache with aura.	eNeura Therapeutics	Neuralieve Cerena Transcranial Magnetic Stimulator	*De Novo*	FDA *De Novo* DEN130022	Linked 510(k): K130556
External trigeminal nerve stimulation	9/15/17	Acute treatment of migraine with or without aura	Cefaly Technology	Cefaly Acute	510(k)	FDA 510(k) K171446	
nVNS	11/27/18	Acute treatment of pain associated with migraine headache; also episodic cluster headache pain	electroCore, Inc	gammaCore Sapphire	510(k)	FDA 510(k) K182369	Also labeled for adjunctive preventive treatment of episodic cluster headache
Remote Electrical Neuromodulation (REN)	1/22/21	Acute treatment of migraine with or without aura	Theranica Bio-Electronics, Ltd	Nerivio	510(k)	FDA 510(k) K203181	Prescription, home-use REN; indicated for migraine (≥12 years)
External trigeminal + occipital nerve stimulation (cranial TENS)	2/16/21	Acute treatment of migraine with or without aura	Neurolief, Ltd	Relivion MG	510(k)	FDA 510(k) K203419	

Representative devices organized by modality and labeled acute-use indication, including acute post-operative/post-traumatic pain (TENS; temporary percutaneous PNS; auricular PENS) and acute primary headache disorders (sTMS; external trigeminal stimulation; noninvasive vagus nerve stimulation; remote electrical neuromodulation; cranial trigeminal + occipital stimulation). “Authorization type” reflects the primary U.S. regulatory pathway [510(k) vs *De Novo*]. Where applicable, the labeled duration of use (e.g., up to 3 days or up to 60 days) is noted. This table is not intended to be exhaustive for device families with multiple substantially equivalent clearances.

a Use statements were summarized and harmonized from FDA marketing authorization documents for comparability and may not reproduce the exact original wording.

**Table 3:** Representative clinical evidence for electromagnetic neuromodulation modalities in pain.

Modality	Pain condition/Population	Methods	Target	Study type/Evidence level	Outcomes	Direction/Size	Safety	Author (first)/Year
SCS	Chronic pain (mixed conditions)	SCS vs conventional medical management (CMM); includes direct and indirect comparisons across SCS types	Spinal cord pathways (dorsal columns/dorsal horn circuits)	Systematic review + network meta-analysis of RCTs	Pain intensity; ≥50% pain responder rate; physical function; health-related quality of life (HRQoL)	All SCS types were superior to CMM for pain intensity at last follow-up (mean difference range −2.37 to −5.55 on 0–10 scale) and for ≥50% pain relief [Odds ratio (OR) range 9.75–63.4]; HRQoL also improved	Safety not quantitatively synthesized; adverse event (AE) reporting was limited/inconsistent across trials	Eldabe 2026 (223)
SCS	Intractable spine and limb pain	Implanted spinal stimulation vs medical therapy (MT); indirect comparison of newer tech vs conventional via common comparator	Spinal cord pathways (dorsal columns/dorsal horn circuits)	Systematic review of RCTs + meta-analysis	≥50% pain relief; pain intensity [visual analog scale (VAS)]	SCS vs MT: ↑ ≥50% pain relief (OR 13.01, 95% confidence interval (CI) 4.96–34.17); VAS ↓ ([weighted mean difference (WMD) 1.43, 95% CI 0.16–2.71). Newer tech (10 kHz/burst/DRG] vs conventional: ↑ pain relief (OR 2.07, 95% CI 1.35–3.19)	AEs mainly hardware/procedure-related; serious neurologic complications were rare, with one subdural hematoma-associated death reported	Lamer 2019 (92)
SCS	Intractable spine and/or limb pain	Implanted SCS vs MT; includes conventional vs high-frequency SCS comparisons (RCTs; min follow-up ≥3 months)	Spinal cord pathways (dorsal columns/dorsal horn circuits)	Systematic review of RCTs + meta-analysis	Opioid/pain medication use (opioid reduction/cessation; Medication Quantification Scale (MQS); morphine equivalent dosage (MED)	Opioid reduction ↑ (OR 8.60, 95% CI 1.93–38.30); MQS ↓ (WMD −1.97, 95% CI −3.67 to −0.27). HF vs conventional SCS: opioid reduction not statistically significant (NS) (OR 1.43, 95% CI 0.74–2.78); MED change NS	AEs variably reported; mostly hardware/implant site pain and lead migration; rare serious neurologic events reported	Pollard 2019 (224)
SCS	Low back pain	Implanted epidural spinal cord stimulation (electrodes + IPG); trials include placebo-controlled cross-over designs and parallel trials adding SCS to medical management	Spinal cord pathways (dorsal columns/dorsal horn circuits)	Cochrane review	Pain intensity, function, quality of life (QoL), AEs (incl. serious AEs (SAEs), need for revision surgery)	6 mo vs placebo: SCS probably does not improve pain/function/QoL (moderate certainty); ≥12 mo mean pain not evaluated. SCS + MT vs MT: pain/QoL uncertain (very low certainty); function/opioid use may slightly improve (low certainty)	Surgical/device AEs (infection, lead migration; occasional SAEs. Revision surgery reported (up to ∼31% at 24 mo in one trial); AE reporting inconsistent across studies	Traeger 2023 (118)
DRG	Complex regional pain syndrome (CRPS) I/II; causalgia (lower extremity focal/dermatomal pain)	Implanted DRG stimulation vs implanted conventional SCS	Dorsal root ganglion → dorsal horn circuits	Implanted DRG stimulation vs implanted conventional SCS (randomized comparative effectiveness trial)	Treatment success (composite incl. ≥ 50% VAS reduction + no stimulation-related neurologic deficit), VAS pain intensity; paresthesia specificity/postural variation	Treatment success higher with DRG vs SCS at 3 mo: 81.2% vs 55.7% (*P* < 0.001); maintained at 12 mo: 74.2% vs 53.0%; less postural variation in paresthesia and less extraneous stimulation in non-painful areas with DRG	Device-related and SAEs not different between groups; typical implant/device risks (e.g., infection, lead migration/lead issues, pocket pain) reported	Deer 2017 (106)
MCS	Refractory chronic neuropathic pain	Implanted epidural motor cortex stimulation with randomized active vs sham crossover comparison	M1	Single center RCT	Pain intensity [numerical rating scale (NRS)]; responder status	Pain reduction during active stimulation was variable; long-term responders were observed in a subset of patients (∼39% long-term responders), but overall efficacy was heterogeneous	Invasive neurosurgical/device-related risks, including infection, seizures, and hardware complications; no major unexpected safety signal in this small trial	Hamani 2021 (52)
VNS (implantable/invasive)	Chronic pain (mixed conditions)	Implanted cervical VNS	Cervical vagus nerve	Narrative review; invasive-VNS evidence subset	Pain intensity/pain relief	Limited and heterogeneous pain-specific clinical evidence; possible benefit reported in selected conditions, but no firm efficacy conclusions	Implant-related AEs possible	Shao 2023 (225)
VNS (implantable/invasive)	Treatment-resistant fibromyalgia	Implanted cervical VNS	Cervical vagus nerve	Prospective open-label Phase I/II proof-of-concept trial	Pain intensity; patient global improvement; physical function; widespread pain and tender-point criteria	Preliminary improvement was reported in a subset, with additional responses during follow-up; interpretation is limited by the small, uncontrolled design	Implantation-, device-, and stimulation-related AEs occurred; one device required revision and one was later explanted for lack of perceived benefit	Lange 2011 (130)
aVNS	Chronic pain (mixed conditions)	Auricular VNS, transcutaneous or percutaneous, vs sham/control	Auricular branch of the vagus nerve	Systematic review and meta-analysis	Pain intensity/analgesic use	Significant reduction in chronic pain intensity vs. sham/control; greater effect with percutaneous than transcutaneous aVNS	Very well tolerated; mainly mild local AEs	Duff 2024 (132)^a^
n-VNS	Migraine	Noninvasive VNS, including cervical (n-cVNS) and auricular (n-aVNS) approaches, vs sham/control	Cervical vagus nerve/auricular branch of the vagus nerve	Systematic review and meta-analysis of RCTs	Monthly migraine/headache days, pain-free rate within 2 h, ≥50% responder rate, headache intensity, AEs	n-cVNS: ↑ ≥ 50% responder rate (OR 1.64, 95% CI 1.10–2.47); no significant reduction in migraine days or headache days. n-aVNS: ↓ migraine days (mean difference (MD) −1.8, 95% CI −3.34 to −0.26) and ↓ headache intensity (standardized MD (SMD) −0.7, 95% CI −1.23 to −0.17)	n-cVNS was safe and well tolerated; no significant increase in AEs vs sham	Song 2023 (226)
n-VNS	Cluster headache^b^	Noninvasive VNS, mainly cervical n-VNS, vs sham/control	Cervical vagus nerve	Systematic review	Attack frequency, pain intensity, responder rates, AEs	Moderate-to-high-quality evidence suggested benefit in reducing cluster headache frequency and intensity; benefit was clearer in episodic than chronic cluster headache, and heterogeneity prevented meta-analysis	Generally safe and well tolerated in noninvasive studies	Fernandez-Hernando 2023 (227)
tDCS	Chronic pain (mixed conditions)	tDCS vs sham	M1 (most studies)/other cortical targets	Cochrane systematic review of sham-controlled randomized and quasi-randomized trials	Pain intensity, disability, QoL, AEs	Short-term pain reduction vs sham: Standardized Mean Difference (SMD) −0.43 (95% CI −0.63 to −0.22), corresponding to ∼0.82 points on a 0–10 scale or ∼17% reduction; no clear disability benefit; long-term effect not supported. Evidence quality very low, with heterogeneity and possible small-study bias	Mostly minor/transient AEs (e.g., headache, scalp irritation, tingling, redness); no major lasting safety signal reported	O’Connell 2018 (153)
tDCS	Nonspecific chronic low back pain	tDCS (multiple sessions) vs sham	M1	Systematic review and meta-analysis of RCTs	Pain intensity, disability, AEs	No significant post-treatment benefit for pain (SMD 0.378, 95% CI −0.264 to 1.020; *P* = 0.249) or disability (SMD 0.143, 95% CI −0.214 to 0.499; *P* = 0.434); evidence does not support clinical use based on available data	No significant AEs reported	Alwardat 2020 (228)
tDCS	Painful diabetic polyneuropathy	Anodal tDCS over left M1 vs sham; 2 mA, 20 min, 5 consecutive days	M1	Randomized, sham-controlled trial	VAS pain intensity, Clinical Global Impression, pain threshold	Significant pain reduction vs sham, sustained at 2 and 4 weeks	No major safety signal reported	Kim 2013 (161)
rTMS	Chronic pain (mixed conditions)	rTMS vs sham	M1 (most studied). Some studies targeting prefrontal cortex	Cochrane systematic review of sham-controlled randomized and quasi-randomized trials	Pain intensity, disability, QoL, AEs	Small short-term effect overall: SMD −0.22 (95% CI −0.29 to −0.16), corresponding to ∼0.40 points on a 0–10 scale; high-frequency single-dose M1 stimulation showed a somewhat larger short-term effect but still did not clearly meet clinical importance thresholds; no clear disability benefit	Mostly minor/transient AEs; two seizures reported with active rTMS across included studies; AE reporting inconsistent	O'Connell 2018 (153)
rTMS	Neuropathic pain	rTMS vs sham	M1 (most studied)	Systematic review and meta-analysis of RCTs	Pain intensity	rTMS was better than sham in reducing pain; significant analgesic effects were associated with M1 stimulation and high-frequency rTMS (≥5 Hz); no evident difference between single- and multi-session protocols	No significant difference in AEs vs. control; reported adverse effects were generally mild/transient	Jiang 2022 (229)
rTMS	Fibromyalgia	10-Hz rTMS vs sham	M1 and DLPFC	Systematic review and meta-analysis of RCTs	Pain intensity, QoL, depression	10-Hz rTMS reduced pain and improved QoL; subgroup analysis found no significant difference between M1 and DLPFC for pain, although M1 showed a nonsignificant trend toward greater analgesia	No pooled AE analysis reported	Zhu 2023 (230)
Deep TMS	Central neuropathic pain	Deep rTMS (H-coil) vs sham; superficial rTMS comparator	M1	Randomized sham-controlled multicenter crossover trial	Pain intensity	Deep and superficial M1 rTMS both produced analgesic effects of similar magnitude; deep-rTMS effects appeared later but tended to last longer on some secondary outcomes.	Mostly mild-to-moderate AEs (mainly stimulation-site pain and headache); no treatment-related SAEs reported	Bouhassira 2024 (231)
TBS	Chronic pain (mixed conditions; mainly neuropathic pain)	Theta burst stimulation alone or as priming before HF-rTMS	Primarily M1	Systematic review	Pain intensity/pain relief	Some studies reported pain relief, particularly with iTBS of M1 alone or as priming before HF-rTMS, but evidence remains limited and insufficient for firm conclusions	Not systematically reported in review summary	Mussigmann 2025 (232)^a^
tACS	Chronic pain (mixed conditions)	tACS vs sham	Mixed targets (M1, bilateral frontal lobe, occipital lobe)	Systematic review of RCTs	Pain intensity	Insufficient evidence for a conclusive effect overall; findings were mixed across fibromyalgia, low back pain, and migraine	Safety not established through pooled analysis; evidence base remains limited	Chang 2023 (233)
CES	Chronic pain (mixed conditions)	CES vs sham/usual care	Cranial application	Systematic review of RCTs	Pain intensity, function, AEs	Evidence was insufficient/inconclusive for clinically important pain benefit across chronic pain conditions	No SAEs reported; minor symptoms occurred, but AEs reporting was limited/inconsistent	Shekelle 2018 (173)
CES	Fibromyalgia	CES vs sham vs usual care	Earlobe electrodes	RCT	Pain intensity, fatigue, sleep disturbance, functional status	CES was associated with greater decreases in pain and sleep disturbance, with improved functional status vs. sham and usual care over time; fatigue also trended to improvement	Very few AEs reported; no blood-pressure safety signal was observed	Taylor 2013 (178)
TENS	Chronic pain (mixed conditions)	TENS vs sham/placebo	Surface electrodes at the site of pain or over nearby peripheral nerve bundles	Systematic review and meta-analysis of RCTs	Pain intensity during or immediately after TENS	TENS was associated with lower pain intensity during or immediately after treatment vs. placebo; subgroup analyses did not identify pain duration (acute vs chronic) or diagnosis as effect modifiers	No SAEs directly attributable to TENS were reported; AEs were generally mild/infrequent and not significantly different from comparators	Johnson 2022 (234)^a^
TENS	Fibromyalgia	TENS vs sham/placebo	Surface electrodes over painful areas	Cochrane systematic review of RCTs	Pain intensity, fatigue, function, QoL, AEs	Very low-certainty evidence; unable to determine whether TENS improves pain vs. sham	No SAEs clearly attributable; AE reporting limited/inconsistent	Johnson 2017 (235)
TENS	Fibromyalgia	TENS vs sham, no intervention, or active comparators (e.g., exercise, physiotherapy modalities, pharmacologic therapy)	Surface electrodes over painful areas	Systematic review and meta-analysis of controlled clinical trials	Pain intensity, disability, QoL	TENS was associated with ↓ pain (SMD −0.61, 95% CI −1.00 to −0.16), ↓ disability (SMD −0.27, 95% CI −0.41 to −0.12), and ↑ physical QoL (SMD 0.26, 95% CI 0.08 to 0.44)	AEs not systematically reported; no pooled safety analysis	Garcia-Lopez 2024 (236)
PNS	Chronic pain (mixed conditions)	PNS vs comparator/sham/usual care	Peripheral nerves	Systematic review	Pain relief, functional improvement, AEs	For chronic pain, evidence was strongest for refractory peripheral nerve injury (level II), with lower-level evidence for some other indications	Safety generally acceptable, but AEs varied by device, target, and study	Helm 2021 (203)
PNS	Hemiplegic shoulder pain after stroke	Single-lead percutaneous PNS vs usual care	Peripheral nerve target in the shoulder/deltoid region	Single center RCT	Pain intensity, pain interference, QoL, AEs	Greater reduction in pain vs. usual care, maintained at least 12 weeks after treatment	No serious infections; lead dislodgement, retained fragments, pruritus, and transient implant-site pain were reported	Wilson 2014 (237)
PNFS	Localized chronic intractable back pain	Randomized crossover comparison of PNFS stimulation conditions; follow-up in implanted responders	Subcutaneous painful field of the back	RCT	Pain intensity, disability, patient satisfaction, AEs	PNFS improved pain during the randomized crossover phase, and responders who proceeded to permanent implantation maintained significant pain reduction through 52 weeks	Generally well tolerated; device/procedure-related AEs were reported	McRoberts 2013 (238)
SCS + PNS	Brachial plexopathy/focal upper-extremity neuropathic pain	Combined SCS with PNS	Cervical spinal cord + radial nerve (selected peripheral target)	Case report	Pain intensity/medication use/functional status	After loss of benefit from prior SCS alone, addition of radial-nerve PNS was associated with disappearance of background pain, residual breakthrough pain at NRS 2–3/10, reduced analgesic use, and maintained benefit at 6 months; evidence is limited to a single case	No procedural complication was reported in this case; AE characterization is limited by single-case evidence	Choi 2016 (239)
SCS + PNFS	Chronic low back and leg pain	Combined SCS with PNFS	Dorsal columns + painful subcutaneous field	Case series	Overall pain control/pain coverage	Most patients reported better overall pain control and improved coverage with the combination than with either modality alone; in some cases, PNFS was added after SCS alone did not adequately control low back pain	No trial-period complications were reported (including no infections or dislodged/pulled wires); broader implant-related risks remain relevant, but formal AE characterization was limited in this small case series	Bernstein 2008 (212)

For each modality–indication pair, we prioritized higher-level evidence syntheses (Cochrane reviews, systematic reviews, and meta-analyses). When such evidence was limited, inconsistent, or unavailable, we selected representative sham-controlled randomized trials or other illustrative prospective studies, emphasizing studies with clearly described stimulation parameters and quantitative pain outcomes. This table is intended to summarize representative evidence rather than provide an exhaustive systematic inventory.

a The cited study includes chronic, acute, and/or experimental pain populations; this table emphasizes the findings most relevant to chronic pain.

b Includes episodic and chronic cluster headache populations.

To support this broad, cross-modality review, targeted searches were conducted using bibliographic and regulatory sources, including PubMed/MEDLINE, Cochrane reviews, and publicly available FDA documents. Searches combined terms related to pain management, with emphasis on chronic pain and selected acute or episodic pain/headache indications, with modality-specific terms spanning peripheral nerve, spinal cord, dorsal root ganglion, vagal/autonomic, cranial/transcranial, cortical, and deep brain neuromodulation approaches, including implanted and noninvasive modalities where applicable.

Historical, mechanistic, technical, clinical, and regulatory sources were used to contextualize the development, rationale, stimulation paradigms, and current clinical positioning of each modality. Primary sources were prioritized for foundational concepts, historical milestones, and specific clinical or experimental findings. Reviews, guidelines, evidence syntheses, and regulatory sources were used to support broader summaries of clinical evidence, regulatory status, mechanistic frameworks, evidence strength, and limitations across modalities. Clinical evidence discussion prioritized systematic reviews, meta-analyses, Cochrane reviews, randomized controlled trials, and selected clinical studies relevant to pain, emphasizing pain-related outcomes, safety, durability, and limitations. Because the goal was to provide a narrative review rather than a systematic review, searches were not intended to be exhaustive, and no formal risk-of-bias or evidence-grading framework was applied.

## 2 Implanted neuromodulation approaches

### 2.1 MCS: historical development of motor cortex stimulation

MCS, typically delivered as epidural brain stimulation (EBS) over M1, involves epidural placement of electrodes over the dura covering M1 to modulate downstream pain networks. Although MCS was formally introduced clinically in 1991 (39), the concept emerged earlier from experimental observations and from efforts to identify cortical targets that might provide analgesia when other central stimulation approaches showed limited success (40). Tsubokawa et al. first reported motor cortex stimulation for pain control in patients with central pain in 1991 (39). In 1993, Meyerson et al. (41) described the use of MCS in different forms of neuropathic pain. In 1995, Migita et al. (42) and Peyron et al. (43) independently reported further evidence of MCS efficacy in central pain studies, with Migita also suggesting that response to TMS over M1 might help predict response to implanted MCS. Over time, implantation and targeting strategies evolved from early epidural approaches toward more refined localization using neuronavigation, intraoperative electrophysiology, and, in some centers, functional imaging (44, 45). Programming, however, remained largely empirical, with stimulation parameters and electrode-contact configurations adjusted according to individual pain response (40, 46). By 2020, a systematic review and meta-analysis for orofacial pain noted that MCS had been performed in approximately 700 cases worldwide for a variety of refractory neuropathic pain syndromes (47).

Mechanisms of analgesia from MCS remain incompletely established, but converging electrophysiological and functional imaging findings support a distributed network-level effect rather than a purely local cortical action. MCS appears to engage thalamic circuitry, together with slower, longer-lasting modulation of medial pain-network regions, including the anterior cingulate and orbitofrontal cortices, as well as midbrain structures such as the PAG (38, 48, 49). Proposed mechanisms include altered affective appraisal of pain through cingulate-orbitofrontal modulation, recruitment of descending inhibitory pathways, and possible involvement of endogenous opioid systems (38, 48).

Clinical evidence for MCS remains largely based on case series and observational cohorts, with relatively few controlled trials and substantial heterogeneity in patient selection and outcome reporting. Analgesic benefit has been reported in selected patients with refractory neuropathic pain, but response rates and durability vary across studies, and overall confidence is limited by small samples, inconsistent follow-up, and limited sham-controlled evidence (47, 50–52).

Stimulation has been delivered using several epidural electrode designs, most commonly 4-contact paddle electrodes, as described in technical and review literature on MCS, and, in some reports, newer 8-contact leads developed specifically for this application (40, 53). Reported programming approaches include monopolar or bipolar/anode-cathode configurations, with contact assignment and amplitude adjusted according to clinical response and tolerability. Published stimulation parameters vary widely, with reported ranges of approximately 0.5–10 V, 5–130 Hz, and 60–450 µs, underscoring the lack of a standardized programming paradigm across studies (40, 46). Daily duration and overall stimulation exposure are not reported consistently; when described, stimulation is often delivered in cycling mode rather than continuously, with on-times ranging from 10 min to 3 h and off-times from 15 min to 6 h (46). Overall, the evolution of MCS reflects a broader challenge in neuromodulation, in which the ability to influence cortical and subcortical networks has not consistently translated into predictable or durable clinical benefit partly because dosing parameters and programming remain heterogeneous (40). MCS is therefore generally reserved as a specialized neurosurgical option for carefully selected patients with refractory neuropathic pain, with broader adoption limited by the scarcity of high-quality controlled evidence (54, 55).

### 2.2 DBS: early intracranial analgesia and evolving targets

DBS involves the direct implantation of stimulating electrodes in selected brain targets to modulate pain processing and has been explored for refractory chronic pain for several decades. Early DBS technology emerged through adaptation of implantable cardiac pacing systems, with Medtronic playing a central role in translating pacemaker-derived neurostimulation hardware into chronic brain stimulation platforms (56). Temporary intracranial stimulation relevant to pain had already been explored in the 1950s (57–60), and chronic implanted DBS approaches for pain were reported in the 1970s (11–13, 61), spurred in part by rodent microstimulation experiments in the late 1960s (10). Initial studies focused on treating intractable pain secondary to cortical injury, anesthesia dolorosa, post-cancer pain, and diabetic neuropathic pain via direct stimulation of the internal capsule (IC), sensory thalamus, or periventricular and periaqueductal gray matter (PVG/PAG) (11, 12, 62–65). Early electrodes were mainly custom-made, featuring a diverse range of materials and dimensions, including's Heath's stainless steel or silver depth probes measuring up to ∼2.0–3.0 mm in diameter; Mazars's fine gold/copper Teflon-insulated wires at ∼0.5 mm in diameter; and Hosobuchi's custom platinum-iridium cylindrical contacts measuring 0.5 mm in diameter and 0.5 mm in length [see (66) for a review of electrode characteristics]. Across early pain-DBS series, stimulation parameters varied by target and center, with frequencies ranging roughly from 2 to 100 Hz, pulse widths from about 0.1 to 0.8 ms, and amplitudes commonly reported in the 0.5–8 V range; historical summaries suggest that PVG/PAG stimulation tended to use lower frequencies (for example, around 25–50 Hz), whereas sensory thalamic stimulation was often reported at 50–100 Hz (66).

Early custom pain-DBS systems were later replaced by commercial Medtronic platforms, initially including radio-frequency systems with transcutaneous power transfer and, in later chronic pain trials, first-generation and subsequent Medtronic leads (56, 67). Later Medtronic DBS leads adopted a standardized quadripolar geometry with a diameter of approximately 1.27 mm, four 1.5-mm-long contacts, and model-dependent edge-to-edge electrode spacing: 1.5 mm for the 3387 and 0.5 mm for the 3389 (68, 69). During this commercial expansion of DBS in the 1990s and 2000s, implanted pulse generators were largely constant-voltage systems. Typical therapeutic programming used amplitudes of about 1–3.5 V, pulse widths of 60–210 μs, and high-frequency stimulation commonly around 130 Hz and extending to 185 Hz; battery longevity depended strongly on the selected stimulation parameters and device circuitry. Because these systems were voltage-controlled, changes in electrode-tissue impedance could alter the actual current delivered and the resulting extent of neural activation (70–73).

Beginning in the mid-2010s, DBS hardware evolved from conventional ring-contact electrodes toward directional segmented leads that enabled current steering. Early commercial directional systems included 8-contact leads with segmented middle levels that could be independently activated to shape the stimulation field more selectively than traditional omnidirectional leads (70, 74). Although this development was first established in the broader DBS field, directional leads have also been used in selected chronic neuropathic pain applications, particularly sensory thalamic DBS (75–77). Boston Scientific's Vercise platform was among the earliest commercial DBS systems to implement multiple independent current control (MICC) (78), a constant-current architecture that allows independent current distribution across contacts, supports more flexible stimulation-field shaping, and can reduce impedance-related fluctuations in delivered stimulation compared with voltage-controlled systems (73). More recent systems have extended this trend to higher-contact-count designs, including 16-contact directional leads, further increasing programming flexibility and spatial selectivity (78, 79). Modern platforms also offer broad programming ranges, with pulse widths reaching up to 450 μs in some systems (70). In parallel, sensing-enabled and biomarker-driven neuromodulation studies have strengthened the rationale for adaptive DBS in chronic pain, but this remains investigational rather than an established clinical standard in pain DBS (80, 81).

Early patient investigations suggested that intermittent rather than sustained stimulation could be more effective, as continuous stimulation was associated with waning efficacy and possible tolerance to narcotic medications (12). Subsequent clinical work shifted toward evaluating long-term efficacy across different pain etiologies and anatomical targets. A meta-analysis of studies published between 1977 and 1997 found that long-term outcomes depended strongly on both pain type and stimulation target: nociceptive pain showed better long-term success than deafferentation pain (63% vs. 47%), and the highest success rates were reported with PVG/PAG stimulation alone (79%) or combined PVG/PAG plus sensory thalamus/internal capsule stimulation (87%) (82). Sensory thalamic targets were used particularly for neuropathic pain, whereas PVG/PAG stimulation was favored in nociceptive pain and linked to endogenous opioid-mediated mechanisms (12, 64, 65, 82). Despite these refinements, durability remained a major limitation, as some patients lost benefit over time and the pivotal Medtronic multicenter pain trials failed to meet prespecified efficacy thresholds. Specifically, the Model 3380 study enrolled 196 patients and the Model 3387 study enrolled 50 patients, but neither achieved the criterion of at least 50% pain relief in half of implanted patients at 1 year (67). Thus, early enthusiasm for DBS in chronic pain was limited by inconsistent long-term outcomes and failure to meet efficacy thresholds in multicenter trials. The lack of FDA-approved DBS indications for chronic pain, together with limited and region-specific European use, underscores a recurring pattern in neuromodulation in which a strong mechanistic rationale does not consistently translate into durable clinical benefit. As a result, DBS remains an off-label or investigational option for chronic pain in most settings, although interest has resurged with recent sensing-enabled and adaptive approaches (80, 81).

Overall, the DBS-for-pain evidence base remains heterogeneous and is still dominated by small case series and observational cohorts spanning diverse pain etiologies and targets, with relatively few modern controlled trials (83). Structured reviews of the multicenter trial experience and subsequent evidence syntheses highlight target- and indication-dependent variability in response and durability, and note limitations related to small samples and inconsistent outcome definitions and follow-up (67, 84). DBS is nevertheless used in highly selected cases, including in post-stroke pain, neuropathy, and pain refractory to prior SCS.

Common stimulation targets include the PVG/PAG, IC, anterior cingulate cortex (ACC), and the ventral posterolateral nucleus and ventral posteromedial nucleus (VPL/VPM), with stimulation delivered using commercial DBS hardware such as Medtronic 3387/3389 leads paired with compatible Medtronic neurostimulators, Abbott Infinity systems, and Boston Scientific Vercise systems with Cartesia directional leads, although stimulation settings in pain DBS remain highly variable across targets and studies.

DBS analgesia is generally considered target-dependent, engaging different components of the pain system (84, 85). Even when initial benefit is achieved, long-term durability remains a major limitation despite advances in hardware and programming. PVG/PAG stimulation is thought to recruit descending pain modulatory circuits, with human data supporting endogenous opioid involvement in at least a subset of patients (e.g., naloxone-reversible effects) (12, 86), although non-opioid and mixed mechanisms have also been reported (87). Sensory thalamic targets (VPL/VPM) are proposed to modulate ascending nociceptive transmission and sensory encoding (84, 85), whereas ACC, particularly dorsal ACC (dACC), stimulation is framed as reducing the affective–motivational burden of pain by modulating limbic–cognitive control networks (88, 89). Across targets, mechanisms remain debated and likely reflect network-level effects rather than a single pathway. Furthermore, although outside the scope of this article, DBS is moving towards closed-loop and personalized dosing and targeting for pain treatment, see (90) for details.

### 2.3 SCS: evolution of dorsal column stimulation and modern waveforms

The evolution of SCS mirrors a broader pattern in neuromodulation, where early physiological models supported rapid clinical adoption, followed by increasing recognition of heterogeneous responses and more complex, network-level mechanisms.

SCS, first introduced in 1967 under the term dorsal column stimulation (91), has evolved considerably as a treatment for intractable chronic pain (92). Its early clinical rationale was closely tied to the gate control theory proposed by Melzack and Wall, which held that activation of large-diameter, non-nociceptive afferents could suppress nociceptive transmission at the dorsal horn (15). After its initial clinical introduction, SCS was progressively formalized through commercial and regulatory development from the 1970s through the early 1990s (93), while parallel technological advances included percutaneous trial stimulation before permanent implantation, adoption of fully implantable pulse generators (IPGs), and development of multichannel systems that expanded programming flexibility and paresthesia targeting (93–96). Later advances in SCS have led to the development of high-frequency (HF) SCS and burst SCS. High-frequency SCS (10-kHz SCS, often referred to as HF10) was developed to reduce or avoid paresthesia associated with traditional SCS by applying a waveform at 10,000 Hz at a subthreshold level (97, 98). Burst SCS delivers stimulation in packets of pulses, classically five pulses per burst, at reduced amplitudes to achieve subthreshold stimulation with little to no paresthesia while maintaining analgesic benefit (99–101).

SCS is delivered by placing electrodes in the epidural space to modulate pain signaling, historically often through paresthesia-based stimulation (102). Temporary percutaneous stimulation before permanent implantation was introduced early in the development of SCS (94), and in current practice patients typically undergo a trial with temporary epidural leads connected to an external pulse generator to assess pain relief and tolerability before placement of a permanent subcutaneous pulse generator (103, 104). If the trial is successful, a permanent subcutaneous pulse generator is implanted to deliver long-term therapy (104). Pulse frequencies may range from approximately 2 to 1,200 Hz, with conventional tonic stimulation frequencies typically in the 40–60 Hz range (104). Pulse magnitudes are patient-specific and are typically programmed in the mA range [commonly across a 0–20 mA device range (105); reported clinical amplitudes for paresthesia-based tonic SCS are often in the low single-digit mA range, e.g., ∼4–9 mA (106)].

Although SCS was initially framed through the gate control theory, contemporary evidence suggests that analgesia likely reflects a combination of segmental spinal effects and supraspinal network modulation, including altered dorsal horn excitability, recruitment of large-diameter afferent fibers, and engagement of descending inhibitory pathways; the relative contribution of these mechanisms may differ across tonic, burst, and high-frequency paradigms (107–111).

Clinically, SCS has been used across selected neuropathic and radicular pain indications, including failed back surgery syndrome, complex regional pain syndrome (CRPS), and painful diabetic neuropathy (112–114). FDA-authorized SCS indications are device-specific, with selected labeling including chronic intractable pain of the trunk and/or limbs and conditions such as failed back surgery syndrome, CRPS, radicular pain, and painful diabetic neuropathy (115, 116). Interpretation of the SCS evidence is also influenced by the common use of trial stimulation before permanent implantation, which may affect patient selection and the composition of implanted cohorts (103). Systematic reviews and network meta-analyses generally support SCS as being associated with improved pain outcomes vs. conventional medical management in selected chronic neuropathic pain populations, although certainty varies by indication, trial design, and outcome assessed (117). In contrast, for chronic low back pain populations studied with placebo/sham comparators, some evidence syntheses have reached more skeptical conclusions, underscoring that comparator choice, patient selection, and follow-up duration can materially influence inferred efficacy (118). SCS has also been reported in post-amputation pain, including phantom limb pain, but the evidence remains limited to small heterogeneous series and a recent scoping review suggesting possible benefit in a subset of patients (119, 120). Cross-study interpretation is complicated by two layers of heterogeneity: tonic, burst, and high-frequency SCS paradigms differ in waveform design, paresthesia profile, and inferred mechanism, and the studies evaluating them also vary in comparator design.

### 2.4 Dorsal root ganglion (DRG) stimulation: targeted stimulation for focal pain syndromes

DRG stimulation emerged as a more anatomically targeted neuromodulation approach within the broader SCS family, designed to improve coverage of focal pain distributions that may be difficult to treat with conventional dorsal column stimulation (121, 122). By placing leads near the dorsal root ganglion, DRG stimulation enables more selective modulation of sensory signaling at the level of the affected dermatome and has been particularly applied in conditions such as CRPS and focal causalgia (106, 121). Because the dorsal root ganglion contains the cell bodies of primary sensory neurons and has been implicated in pathological sensory signaling in chronic pain states, it represents a biologically relevant target for neuromodulation (121). This anatomical specificity may be particularly advantageous in focal neuropathic pain confined to discrete dermatomal or localized regions (106, 123). Compared with conventional SCS, DRG stimulation may offer more stable paresthesia coverage in anatomically discrete pain regions, although programming remains individualized and evidence varies by indication (106, 122, 123). In the United States, DRG stimulation received FDA approval in 2016 for neuropathic pain associated with CRPS and causalgia of the lower extremity (124, 125), and the strongest comparative clinical evidence remains concentrated in these focal neuropathic pain populations (106). Overall, DRG stimulation represents the move in the neuromodulation field toward anatomically precise targeting. Nevertheless, the variability in clinical response highlights that increased spatial specificity does not fully resolve the physiological puzzle of pain network modulation.

## 3 Vagal neuromodulation

### 3.1 VNS: evolution of implanted and noninvasive approaches

VNS targets vagal afferent pathways to modulate central pain networks and autonomic-neuroimmune signaling (126, 127). Although VNS was first clinically evaluated as an implanted cervical therapy for refractory epilepsy (128) and later for treatment-resistant depression (129), implanted cervical VNS has also been explored in treatment-resistant fibromyalgia (130). More recently, noninvasive VNS approaches have emerged, including cervical nVNS for headache disorders (131) and auricular VNS for acute and chronic pain conditions (132, 133). Accordingly, this subsection considers both implanted and noninvasive VNS approaches.

Clinically, VNS can be delivered invasively via an implanted cervical vagus nerve stimulator or noninvasively via transcutaneous auricular VNS (taVNS) or cervical nVNS (126, 134). Although these approaches are often grouped under the VNS umbrella, implanted cervical VNS, cervical nVNS, and taVNS differ substantially in hardware, dosing, anatomical access, and current clinical positioning (126, 134, 135). In pain medicine, the strongest regulatory and clinical support relates to cervical nVNS for headache disorders, whereas implanted VNS and transcutaneous/auricular VNS applications in non-headache chronic pain remain more exploratory and are supported by a heterogeneous evidence base (132, 134, 136).

Dosing and programming vary by device and indication. In taVNS clinical studies, commonly reported settings include ∼25 Hz stimulation with a pulse width of ∼0.5 ms, with intensity titrated to the maximum tolerated sensory level (often within the low-mA range), sometimes delivered in duty cycles for session-based use (137). For cervical nVNS used for headache indications, dosing is typically delivered as short, repeated ∼2-minute applications per treatment episode (acute use), with preventive regimens depending on the labeled indication (138, 139).

Mechanistically, VNS is thought to engage vagal afferent pathways projecting to brainstem relay nuclei, particularly the nucleus tractus solitarius, with downstream effects on networks involved in ascending pain processing and descending inhibitory control. VNS has also been linked to autonomic and neuroimmune pathways, although the relative contribution of these mechanisms may differ across implanted cervical, noninvasive cervical, and auricular approaches (127, 133). A recent randomized, sham-controlled trial in rheumatoid arthritis further supports the clinical relevance of vagus-mediated neuroimmune modulation, showing that implanted cervical VNS can modulate inflammatory disease activity in humans. These findings support target engagement of the inflammatory reflex, although they do not directly establish analgesic efficacy in chronic pain (140).

Evidence for VNS in pain is indication-dependent. nVNS has regulatory clearance for certain headache indications, including migraine and cluster headache (138), and has also been studied in other chronic pain conditions with heterogeneous results. A recent systematic review and meta-analysis of transcutaneous VNS reported measurable pain reduction across some chronic pain conditions, but emphasizes variability in populations, stimulation parameters, comparators, and outcomes (136). Overall, the development of VNS embodies the expansion of neuromodulation beyond traditional somatosensory networks. Its heterogeneous outcomes underscore the challenges of translating autonomic/neuroimmunological modulation into consistent analgesic effects in the context of different stimulation sites (auricular vs. cervical), dosing schedules, and comparator conditions. Evidence is strongest for labeled headache indications, whereas magnitude and durability of benefit in non-headache chronic pain conditions remain uncertain and appear sensitive to protocol and population differences (134, 136, 138, 139).

## 4 Noninvasive central/cranial neuromodulation

### 4.1 TMS: from noninvasive cortical stimulation to analgesic protocols

TMS was developed in the mid-1980s as a noninvasive method for stimulating the human cortex (14) through the intact scalp and was later extended from single-pulse neurophysiologic applications to repetitive and patterned therapeutic protocols (141, 142). TMS uses electromagnetic coils to generate brief, rapidly changing magnetic fields that induce electric currents in neural tissue, thereby activating cortical neurons and modulating neural excitability (2, 14, 143). TMS can produce pulsed fields of up to 4 Tesla, but commonly near 2 T, with pulse durations on the order of approximately a millisecond for single-pulse stimulators (with shorter rise times) and approximately a quarter of a millisecond duration for rapid stimulators (14, 144, 145). Stimulation effects can be inhibitory or excitatory depending on the frequency and pattern of stimulation and can persist beyond the duration of stimulation (143). The most widely used therapeutic form, rTMS, is typically delivered with a figure-of-eight coil and primarily reaches superficial cortical targets (typically 2–3 cm in depth). Deep TMS, which is postulated to reach deeper structures than conventional rTMS, albeit with reduced focality, can be delivered with different coils, such as double-cone, Halo, and H-coils (146). Finally, theta-burst stimulation (TBS) is a patterned form of rTMS in which short high-frequency bursts (classically 3 pulses at 50 Hz) are repeated at a theta rate (classically 5 Hz) and delivered in continuous (cTBS) or intermittent (iTBS) patterns (147). In M1 physiology studies, cTBS is often associated with reduced and iTBS with increased corticospinal excitability, although direction and duration can vary with protocol and context (147). TBS over M1 has also been explored in pain indications within the broader evidence base for therapeutic rTMS (141).

Both rTMS and deep TMS have been investigated for pain modulation, with the bulk of research focused on rTMS. The most studied target of stimulation is the motor cortex, but other targets, such as the dorsolateral prefrontal cortex (DLPFC) or the parietal cortex, have also been explored (141, 148–150). Neuropathic pain syndromes have been shown to be among the conditions most responsive to TMS (142, 151). In this patient population, stimulation applied to the contralateral side of pain has been shown to induce analgesic effects; different therapeutic protocols have been explored with various stimulation parameters, such as frequency (e.g., 10 Hz or 20 Hz); intensity [expressed as a percentage of the resting motor threshold (RMT)]; number of pulses (1,500–3,000); and number of sessions [see (141, 142) for a more complete list of stimulation parameters]. A typical protocol used by many laboratories consists of 10 consecutive weekday sessions followed by maintenance sessions at 1 week, 2 weeks apart, and a month apart (142). All of the above parameters might influence the magnitude and duration of pain relief (152).

The mechanisms underlying the analgesic effect of TMS remain incompletely established, but TMS is thought to modulate the activity of cortical and subcortical brain structures that are involved in pain processing (148). While TMS is considered a safe and well-tolerated form of brain stimulation, efficacy results have been mixed (153). A recent Cochrane review on rTMS applied to M1 showed that the analgesic effects of neither single-session nor multi-session protocols are likely to be clinically significant or improve disability (153). The mixed efficacy observed with TMS reflects a recurring challenge in neuromodulation, where heterogeneity among studies remains high and subsequently the ability to modulate neural circuits does not consistently translate into conclusive results pertaining clinical efficacy, optimal target site, and optimal dosing (152).

### 4.2 Transcranial direct current stimulation (tDCS): from cortical polarization to analgesic montages

Although weak transcranial direct current stimulation had been explored in human studies in the 1960s and 1970s, contemporary tDCS was revitalized in the early 2000s after reproducible demonstrations of polarity-dependent modulation of cortical excitability and has since been increasingly investigated as a noninvasive approach for pain modulation (154, 155).

tDCS uses electrodes placed on the scalp to deliver low-intensity direct current to underlying cortical targets, thereby modulating cortical excitability and influencing pain-related networks. tDCS typically delivers 1–2 mA of constant direct current through electrodes of ∼25–35 cm^2^ for 20 min/session (156), increasing or decreasing excitability of neural targets depending on the relative electrode orientation (i.e., field polarity relative to neural targets) (154). When stimulation is provided over multiple sessions, more persistent network effects can be induced; for chronic pain therapy, stimulation is typically delivered in repeated 20-min sessions, often over 5–10 sessions.

tDCS has been used to target different conditions by using different montages. There are two main approaches for tDCS modulation of chronic pain, either placing the electrodes over M1, thereby modulating plasticity of motor circuits related to pain (see MCS above), or over the DLPFC to impact cognitive–affective control of pain (e.g., attentional control, reappraisal, catastrophizing) (157, 158). These montages reflect two complementary therapeutic frameworks: M1 stimulation is generally interpreted as engaging sensorimotor and descending modulatory pathways, whereas DLPFC stimulation is used to influence cognitive–affective dimensions of pain. In both cases, analgesic effects are thought to arise from modulation of distributed pain-related networks rather than direct focal suppression of nociceptive input (157, 158). These network-level effects are thought to involve distributed cortico-subcortical networks, including frontostriatal and descending modulatory pathways, with putative interactions with reward- and brainstem-modulatory nodes (e.g., the nucleus accumbens and PAG). Sham-controlled trials have reported short-term analgesic effects of anodal M1 tDCS (2 mA, 20 min, 5 consecutive days) in fibromyalgia (159, 160), central neuropathic pain after traumatic spinal cord injury (160), and painful diabetic polyneuropathy, with benefits persisting for weeks in follow-up (161). However, across indications the literature remains heterogeneous in populations, dosing, sham methods, and outcomes, and a 2018 Cochrane review concluded that “very low-quality evidence suggests that tDCS may have short-term effects on chronic pain” (153). These findings further illustrate the gap between physiologically plausible modulation of neural networks and reproducible clinical benefit, with evidence suggesting modest, short-term analgesic benefit in selected conditions, but durability and optimal montage/dose remain uncertain due to variability in protocols and outcomes (153).

### 4.3 Transcranial alternating current stimulation (tACS): oscillatory modulation in pain

Human experimental studies of tACS began in the late 2000s, and subsequent work established the technique as an oscillation-oriented form of transcranial electrical stimulation designed to interact with endogenous brain rhythms (162, 163). Similar to tDCS, tACS uses scalp-placed electrodes to apply weak alternating currents noninvasively to cortical targets. tACS delivers a weak (<2 mA) alternating electrical current between conventional scalp electrodes (typically ∼25–35 cm^2^ saline-soaked sponge pads; e.g., 5 × 5 or 5 × 7 cm) (164, 165) and aims to interact with the naturally rhythmic electrophysiologic activity of the brain, which can be detected by electroencephalography (EEG) and magnetoencephalography (MEG). tACS is thought to promote entrainment (i.e., synchronization or desynchronization) of brain oscillations to the stimulation frequency (163), and may also modulate long-range oscillatory coupling between distributed brain regions (e.g., phase-specific changes in interhemispheric functional connectivity) (166). The stimulation frequency can be fixed or matched to specific features of oscillatory activity recorded from the patient (167). As with other transcranial electrical stimulation techniques, the physiological and behavioral effects of tACS likely depend on montage, current intensity, stimulation frequency, session duration, and baseline brain state of the participant, which complicates comparison across studies (164–167). In pain-related studies, montages have typically been designed to target somatosensory or prefrontal regions, although the optimal electrode configuration for engaging pain-relevant oscillatory networks remains uncertain (167–170). Typical experimental montages include bilateral or unilateral primary somatosensory cortex or frontally oriented configurations targeting the dorsolateral prefrontal cortex, sometimes combined with extracephalic reference electrodes (165, 167). In the brain, pain has been associated with oscillations at alpha (8–13 Hz) and gamma (30–100 Hz) frequencies over somatosensory and prefrontal areas (171); these oscillations can be targeted using tACS at the same frequency to promote entrainment (163), which may in turn influence pain perception. This oscillation-based framework provides a compelling mechanistic rationale for the use of tACS in pain modulation, particularly considering evidence that pain-related alterations in alpha and gamma activity occur across somatosensory and frontal networks (171). However, whether these oscillatory signatures are causally related to clinically meaningful analgesia remains uncertain, and the therapeutic value of frequency-matched or individualized tACS protocols has yet to be established (168–170, 172).

To date, relatively few studies have investigated the use of tACS for pain modulation, with mixed results. Clinical evidence remains preliminary and consists largely of small pilot trials with heterogeneous protocols and outcomes, so effectiveness and durability are uncertain and not yet guideline-established (168–170, 172). While some studies have provided preliminary evidence that tACS at alpha frequencies delivered over somatosensory areas during one or more sessions [e.g., 10 sessions over 2 weeks in (172)] may produce analgesic effects (168, 170, 172), other studies using tACS at alpha and gamma frequencies over prefrontal and somatosensory areas have been inconclusive or shown no effect (169).

Overall, tACS provides a mechanistically appealing approach grounded in oscillatory brain dynamics, yet its clinical trajectory reflects the broader challenge of translating modulation of neural rhythms into consistent and clinically meaningful analgesia.

### 4.4 Cranial electrical stimulation (CES): from electrosleep to cranial electrotherapy

CES is a noninvasive neuromodulation technique that applies very low-intensity pulsed electrical currents to the head, commonly via ear-clip (earlobe) electrodes and, less commonly, scalp montages, with the aim of modulating brain networks relevant to pain and related symptoms such as sleep and mood (173). Historically, CES emerged from early 20th-century “electrosleep” approaches, which were intended to induce a sleep- or sedative state, although later investigators argued that any apparent sleep-promoting effects were more likely indirect and related to relaxation rather than true sleep induction (174). Over time, the clinical framing shifted from attempts to induce electrosleep toward broader symptomatic modulation of pain, anxiety, insomnia, and related conditions, such that modern CES is better understood as an evolution rather than a direct continuation of early electrosleep paradigms. CES gained broader clinical use in the United States in the 1960s and 1970s, making it one of the earliest low-intensity cranial electrical neuromodulation modalities to enter broader clinical use (175). CES is often described as a form of transcranial pulsed current stimulation (tPCS) and is technically distinct from tDCS/tACS in that it typically uses pulsed waveforms and can be delivered at low current intensities through infra- or supra-auricular electrode placements (176). Early electrosleep protocols generally used pulsed currents in the 30–100 Hz range (174), whereas contemporary CES devices use device-specific parameters, with commonly reported modern settings including microcurrent intensities and frequencies such as 0.5 or 100 Hz (177). Current systems provide a versatile range of output parameters, with pulse durations ranging from microseconds to milliseconds and current intensities from microamps to upwards of 1 mA. These devices typically utilize biphasic square-wave pulse forms. Proposed mechanisms remain incompletely established and are generally framed in terms of broad modulation of cortical and subcortical network activity, rather than a single established analgesic pathway (177).

CES exemplifies a longstanding gap in neuromodulation in chronic pain between early clinical technological adoption and mechanistic clarity. Systematic evidence assessments have concluded that clinical benefits are uncertain and the evidence base is heterogeneous across populations, comparators, and outcomes (173), although some individual studies in specific conditions such as fibromyalgia have reported improvements in pain and related symptoms (178, 179). Interpretation of the CES literature is further complicated by substantial variability in study design, stimulation parameters, and outcome measures, as well as by the inclusion of earlier studies conducted prior to contemporary trial standards, making it difficult to define a clear therapeutic role for CES in chronic pain management.

## 5 Peripheral neuromodulation

Peripheral neuromodulation includes both noninvasive surface stimulation techniques and implanted or percutaneously placed systems targeting either named peripheral nerves or broader painful fields.

### 5.1 Noninvasive peripheral stimulation

#### 5.1.1 TENS: gate-control foundations and clinical Use

TENS emerged in the early 1970s, shortly after the publication of gate control theory, and became one of the earliest noninvasive electrical therapies used for pain management (15, 180, 181). TENS is delivered across the skin surface to stimulate peripheral nerves through electrode pads. Standard TENS is generally considered safe and inexpensive, typically consisting of a battery-powered hand-held device that generates pulsed electrical currents (182).

Stimulation is characterized by current amplitude, pulse width, and frequency. In clinical use, intensity is typically titrated above sensory threshold to produce a perceptible, non-painful paresthesia. Typical clinical TENS uses frequencies of ∼2–150 Hz and pulse widths of ∼50–400 μs, with sessions commonly lasting ∼20–60 min (183–185). FDA 510(k) summaries for commercial TENS devices report maximum output currents commonly in the tens to low hundreds of milliamperes, with values depending on device and load condition (186–188). The original principle of TENS derives from the gate control theory of pain, which proposes that activation of non-nociceptive afferent input can inhibit transmission of painful signals at the spinal level (15). This is commonly framed as preferential activation of large-diameter, non-nociceptive afferent fibers (182), which may inhibit nociceptive transmission at the spinal level and engage descending inhibitory systems (182). Mechanistic studies have further extended the original gate-control framework to include frequency-dependent neurochemical modulation (e.g., endogenous opioid and serotonergic signaling) and recruitment of descending inhibitory pathways that regulate spinal nociceptive transmission (182, 189–191). These mechanisms reflect convergence of small- and large-diameter sensory inputs onto spinal neurons involved in transmitting sensory information to the brain, together with modulation by descending control pathways (15, 182). Overall, TENS became an established, portable, noninvasive option whose development paralleled the evolving understanding of frequency-dependent neurochemical modulation. Its clinical efficacy, however, has remained inconsistent across pain conditions and study designs. Earlier reviews reached negative or limited conclusions, in part because of methodological limitations and insufficient data suitable for quantitative pooling (192, 193). More recently, Paley et al. conducted a descriptive analysis of 169 reviews (eight overviews, seven hybrid reviews and 154 systematic reviews with 49 meta-analyses), and they recommended TENS as a treatment option for symptomatic relief of pain (194). Their appraisal found that pain intensity was lower during or immediately after TENS compared with control interventions for acute and chronic pain (194). In perioperative settings, TENS has also been evaluated as an adjunct to conventional analgesic management, with evidence suggesting reductions in postoperative pain and analgesic consumption (195). These apparently divergent findings may reflect differences in treatment delivery, because analgesic response depends in part on achieving adequate stimulation intensity and underdosing may contribute to negative or inconsistent trial results (184, 196, 197). Overall, the strongest signals of benefit are observed during active TENS and, in some contexts, in reduced analgesic use, but confidence in broader and longer-term efficacy remains limited by frequent shortcomings in randomized clinical trials (RCTs), insufficient pooled data, and substantial variability across pain conditions and outcomes (194).

### 5.2 Implanted and percutaneously placed peripheral stimulation

#### 5.2.1 Peripheral nerve stimulation (PNS): from surgical implants to percutaneous systems

PNS has been used for pain control for more than five decades, evolving from surgically implanted systems to modern percutaneous approaches. Early clinical work by Shelden and colleagues (198) reported temporary suppression of trigeminal neuralgia pain using implanted PNS electrodes (including high-frequency stimulation delivered via an implanted receiver). In 1967, Wall and Sweet described pain suppression during stimulation in a series of eight patients with chronic cutaneous pain (199). Historically, PNS required open surgical dissection to visualize the nerve and position the lead (200), and subsequent surgical modifications (e.g., interposing a fascial graft between lead and nerve) were later deemphasized due to complexity and complications (201). The modern shift toward percutaneous PNS was catalyzed in 1999 by Weiner and colleagues, who reported that percutaneous lead placement provided analgesia in occipital neuralgia (202). PNS involves placement of a stimulating lead adjacent to a specific peripheral nerve, typically percutaneously under image guidance, to deliver electrical pulses intended to reduce pain. Although mechanisms are not fully established, PNS is generally thought to reduce pain through activation of non-nociceptive afferent fibers and modulation of peripheral and spinal excitability, with possible downstream effects on central sensitization and broader pain network processing (203, 204).

The evolution of PNS reflects a broader trend toward less invasive and more anatomically targeted approaches. The persistent variability in outcomes highlights the ongoing challenge of translating peripheral modulation into consistent therapeutic effects within larger nociceptive networks. Evidence-based clinical guidelines and systematic reviews support PNS as an option for selected acute and chronic pain conditions, although the strength of evidence varies by indication and target nerve (205). However, across indications the literature remains heterogeneous in trial design, comparators, and follow-up duration, and higher-quality controlled evidence is stronger for some targets and conditions than others (205). Dosing/programming is individualized by target and system; example settings reported in clinical studies include pulse widths around ∼200 μs and frequencies around ∼100 Hz, with amplitude titrated to a comfortable sensory percept (paresthesia) (206).

#### 5.2.2 Peripheral nerve field stimulation (PNFS): development of field-based stimulation

Whereas PNS targets a specific named peripheral nerve, PNFS evolved as a field-based strategy for regional pain distributions that are less amenable to focal nerve targeting. Field-based subcutaneous peripheral stimulation approaches were described in the early 2000s under related terms, including targeted subcutaneous stimulation and peripheral nerve field stimulation. Early reports described targeted subcutaneous stimulation and PNFS for neuropathic, abdominal, axial, and chronic low back pain; these case reports and small case series were later followed by larger observational reports that helped define PNFS as a distinct neuromodulation approach (207–211).

PNFS involves placement of leads within the subcutaneous tissues of the painful region to create a “field” of stimulation, rather than targeting a named peripheral nerve as in conventional PNS (209). It has been used both as stand-alone therapy and as an adjunct to SCS in patients with regional pain distributions (212). This field-based approach may be advantageous when a discrete peripheral nerve target cannot be identified. Although mechanisms are not fully established, PNFS is generally thought to modulate afferent input from the painful region by stimulating local nerve branches and cutaneous afferents, with downstream convergence at the spinal cord (209). It has most commonly been applied to localized or regional pain syndromes, particularly chronic axial low back pain. Stimulation is programmed in a patient-specific manner, typically titrated to comfortable paresthesia when paresthesia-based paradigms are used (209). In chronic low back pain, prospective multicenter observational data suggest improvements in pain and function, though study designs and comparators vary (213). A recent narrative review of PNFS for chronic low back pain and persistent spinal pain syndrome similarly concluded that PNFS, used alone or as an adjunct to SCS, appears to reduce pain and analgesic use and improve physical function, although high-quality long-term evidence remains limited (214). Overall, PNFS reflects a shift towards less invasive yet targeted peripheral approaches. However, its heterogeneous outcomes underscore the difficulty of achieving consistent analgesia across variable presentations.

## 6 Hybrid, multimodal, and dual energy approaches

Hybrid neuromodulation refers to pairing two stimulation modalities to engage complementary nodes or mechanisms within the pain system (including combinations of central and peripheral approaches), two peripheral strategies, or multimodal central stimulation. At present, hybrid approaches are better understood as an emerging design direction in pain neuromodulation than as a mature evidence class. In practice, such combinations are most often explored when a single modality provides incomplete relief or insufficient coverage of a complex pain distribution. The mechanistic rationale is that hybrid modalities may allow modulation of complementary peripheral, spinal, and supraspinal components of pain processing, although cumulative effects remain incompletely established. Clinically, most hybrid strategies remain exploratory and are typically used to address incomplete coverage or partial response, such as combining SCS with PNFS for axial low back and leg pain (212), or combining tDCS with peripheral stimulation in chronic pain (215, 216). While not classically an electrical approach, emerging central approaches may also be considered within this framework, including electrosonic stimulation, which combines tDCS with transcranial ultrasound (217, 218). Many hybrid and multimodal neuromodulation approaches recognize that single-target strategies may be insufficient to address the distributed and plastic nature of pain networks. Overall, the published literature on hybrid and multimodal approaches remains limited and heterogeneous, with variability in study design, stimulation protocols, and patient selection (215).

To complement the regulatory summaries in Tables 1–2, Table 3 synthesizes representative clinical evidence across the major electromagnetic neuromodulation modalities discussed in this review. Table 4 summarizes typical stimulation characteristics across these modalities.

**Table 4:** Typical stimulation characteristics of major electromagnetic neuromodulation modalities used in pain research and clinical practice.

Modality	Typical target/Placement	Stimulation type	Usual treatment pattern	Key caveat
SCS	Epidural space over dorsal columns	Implanted electrical stimulation	Trial stimulation followed by permanent implant if successful; can be continuous or programmed use	Parameters vary widely across tonic, burst, HF10, and other paradigms
DRG	Lead positioned near dorsal root ganglion	Implanted electrical stimulation	Trial followed by implant in selected focal pain conditions	Best considered a specialized SCS variant rather than a wholly separate platform
DBS	Intracranial pain-network targets (e.g., PAG/PVG, thalamus, ACC)	Implanted electrical stimulation	Implanted therapy, historically often intermittent daily use; modern use individualized	Highly target-dependent and heterogeneous across studies
MCS	Epidural electrodes over M1	Implanted electrical stimulation	Usually chronic implanted therapy with individualized programming	Dosing and daily use schedules are inconsistently reported
Implanted VNS	Cervical vagus nerve	Implanted electrical stimulation	Chronic implanted therapy	Pain evidence is less established than for epilepsy/depression indications
nVNS/taVNS	Cervical vagus nerve externally or auricular vagal territory	Transcutaneous electrical stimulation	Acute-use or repeated session-based protocols depending on indication	Parameters and targets vary substantially across cervical vs auricular approaches
tDCS	Scalp electrodes over M1 or DLPFC	Weak direct current	Usually repeated over 5–10 sessions	Montage and total dose vary across studies
rTMS	Superficial cortical targets, most commonly M1; also DLPFC or parietal cortex	Noninvasive magnetic stimulation	Often delivered over repeated weekday sessions, sometimes with maintenance sessions	Coil type, target, frequency, and maintenance schedule differ widely
Deep TMS	Deeper and broader cortical/subcortical targets depending on coil design	Noninvasive magnetic stimulation	Repeated session-based protocols	Potentially greater depth with reduced focality; pain evidence remains limited
TBS	Usually M1	Patterned rTMS (bursts at theta rate)	Short-session protocol, sometimes repeated over multiple visits	Pain evidence is more limited than for conventional rTMS
tACS	Scalp electrodes over cortical targets, often somatosensory/prefrontal regions	Weak alternating current	Single- or multi-session protocols	Early-stage field with limited standardization
CES	Ear-clip (earlobe) electrodes, less commonly scalp	Low-intensity pulsed cranial current	Repeated session-based use	Parameters are highly device-specific and evidence remains heterogeneous
TENS	Surface electrodes over painful region or related peripheral nerve territory	Transcutaneous pulsed electrical stimulation	Often 20–60 min/session, repeated as needed	Effectiveness is sensitive to intensity, adherence, and timing relative to pain
PNS	Lead adjacent to named peripheral nerve	Implanted/percutaneous electrical stimulation	Usually trial or staged percutaneous/implant therapy depending on system	Highly target- and system-specific
PNFS	Subcutaneous leads within painful field	Field-based electrical stimulation	Chronic implanted or percutaneous use in selected cases	Less standardized than named-nerve PNS

## 7 Discussion

The evolution of neuromodulation in pain reflects the constant tension between mechanistic understanding and technological prowess. Initial basic physiology and pathophysiology knowledge historically triggered advances in device precision, which often outpaced the identification of more complex anatomical and network-level mechanisms needed to achieve consistent clinical efficacy. A central theme of this review is the divergence between the relatively narrow set of FDA-cleared or approved indications and the much broader published literature spanning additional pain conditions, targets, stimulation paradigms, and outcomes. This distinction matters when interpreting clinical claims, because a larger literature does not necessarily imply regulatory alignment, evidentiary maturity, or translational readiness. Despite advances in device development, anatomical targeting, and regulatory translation, important evidence gaps persist.

### 7.1 Evidence gaps across electromagnetic neuromodulation modalities

The strength and maturity of evidence remain uneven across electromagnetic neuromodulation approaches. The contrast between FDA-cleared or approved indications summarized in Tables 1, 2 and the broader representative evidence base summarized in Table 3 highlights an important translational gap: many modality-indication pairs remain supported by limited, heterogeneous, or non-definitive evidence despite substantial published literature. Although this narrative review does not apply a formal evidence-rating framework, this organization provides a narrative assessment of the relative maturity, limitations, and translational readiness of the available literature.

Several recurring evidence gaps can be grouped into methodological, dosing/programming, comparator selection, sham credibility, blinding, outcome, and technical domains. Methodologically, many studies are limited by small sample sizes, short follow-up durations, and heterogeneous patient populations without adequate stratification. These limitations make it difficult to determine whether inconsistent results reflect true differences in efficacy, differences in patient selection, or variability in trial design. With respect to dosing and programming, stimulation parameters are highly variable and often incompletely reported. The modality-specific differences summarized in Table 4 further illustrate the importance of clearly reporting targets, stimulation parameters, treatment schedules, and implementation details. Many studies do not clearly distinguish the nominal, protocol-specified regimen from the treatment actually administered, which may vary because of titration, placement variability, adherence, session completion, and protocol deviations.

Comparator selection, sham credibility, and blinding limitations may also complicate interpretation across both noninvasive and implanted interventions. In neuromodulation trials, active stimulation can produce sensory, auditory, procedural, autonomic, or device-related cues that are difficult to reproduce in sham conditions. In rTMS, many sham methods have historically failed to reproduce the look, sound, and somatosensory experience of active stimulation while avoiding delivery of a significant magnetic field (219). For tDCS, brief ramp-up stimulation followed by discontinuation at the beginning and end of treatment sessions can produce comparable early cutaneous sensations and support blinding under specific stimulation parameters (220). In implanted neuromodulation, particularly SCS, comparator selection, sham design, and blinding are also challenging because paresthesia, device programming, patient interaction with implanted hardware, and incomplete reporting of sham procedures can affect interpretation (221). Because pain outcomes are subjective and sensitive to expectations, placebo analgesia and nonspecific procedural effects can be difficult to separate from stimulation-specific effects in neuromodulation trials (222). Inadequate sham control or incomplete blinding may therefore inflate apparent treatment effects when participants can infer treatment assignment. These issues limit interpretability when comparator methods, sham credibility, and blinding integrity are incompletely reported or not formally assessed.

Outcome heterogeneity is another recurring limitation, as trials differ substantially in their assessment of pain intensity, function, quality of life, sleep, mood, medication use, and durability of benefit. This variability makes it difficult to compare modalities or determine whether short-term pain reduction translates into sustained clinical benefit. Additional technical limitations include limited precision in target engagement, insufficient mechanistic biomarkers, incomplete personalization of stimulation parameters, and limited development of adaptive or closed-loop control strategies. Together, these methodological, dosing/programming, comparator, sham, blinding, outcome, and technical gaps make it difficult to draw firm conclusions about efficacy, durability, comparative effectiveness across modalities, or which patient populations are most likely to benefit.

### 7.2 Future directions

Future work should focus on adequately powered multi-center trials with standardized reporting of stimulation parameters, longer follow-up to evaluate durability and tolerance, and harmonized core outcomes that include function and quality of life. To improve interpretability and enable cross-study comparison, future trials of electromagnetic neuromodulation for pain should report, at minimum, the following, organized by domain:

*Population and condition*: The pain condition and diagnostic criteria, and patient characteristics relevant to treatment response (e.g., pain phenotype, duration, prior therapies).

*Stimulation and delivery*: The stimulation target and localization method, device type, planned (nominal) stimulation parameters, session number and duration, and overall treatment schedule. Critically, trials should also report the treatment actually delivered, distinguishing it from the nominal protocol, since titration, parameter adjustment, adherence, session completion, and protocol deviations can cause planned and delivered treatments to differ.

*Comparator and blinding*: The comparator or sham procedure in sufficient detail to judge its credibility, and whether and how blinding was assessed.

*Clinical and safety outcomes*: Prespecified pain intensity, function, quality of life, medication use, and adverse events, each with follow-up duration adequate to assess durability. Where feasible, trials should report not only pain and function but whether functional gains track analgesic gains, as these two outcomes may diverge.

*Modality-specific reporting*: For invasive approaches, implantation and procedural variables (lead type and position, target verification). For noninvasive approaches used repeatedly outside the clinic, particularly transcutaneous or home-capable modalities, studies should report placement verification, intensity-adjustment procedures, adherence to the prescribed schedule, and device-recorded usage logs where available.

Improved phenotyping and prespecified subgroup analyses are needed to refine patient selection for specific interventions. Mechanistic and target-engagement measures, such as sensory profiling, autonomic metrics, and neurophysiology, may also help optimize stimulation parameters and complement patient-reported and functional outcomes by assessing whether the intended biological target is being engaged. Future studies should define more clearly where each modality fits within the clinical care pathway, including integration with multimodal pain management, long-term safety and maintenance considerations, and practical issues of implementation, access, and reimbursement. Lastly, emerging adaptive and closed-loop neuromodulation systems represent an effort to address a central limitation of earlier approaches (i.e., the lack of real-time feedback linking stimulation delivery to physiological effect and clinical response). The addition of sensing capabilities and biomarker-driven adjustment of stimulation parameters may help bridge the gap between mechanistic modulation and consistent therapeutic efficacy.
